# Current Targeted Therapies for the Fight against Non-Small Cell Lung Cancer

**DOI:** 10.3390/ph13110374

**Published:** 2020-11-09

**Authors:** Lisa Maria Mustachio, Jason Roszik

**Affiliations:** 1 Department of Epigenetics and Molecular Carcinogenesis, The University of Texas MD Anderson Cancer Center, Houston, TX 77030, USA; 2 Center for Cancer Epigenetics, The University of Texas MD Anderson Cancer Center, Houston, TX 77030, USA; 3 Department of Genomic Medicine, Division of Cancer Medicine, The University of Texas MD Anderson Cancer Center, Houston, TX 77030, USA; 4 Department of Melanoma Medical Oncology, Division of Cancer Medicine, The University of Texas MD Anderson Cancer Center, Houston, TX 77030, USA

**Keywords:** NSCLC, targeted therapy, clinical trials, ALK, EGFR, ROS1, BRAF

## Abstract

Lung cancers contribute to the greatest number of cancer-related deaths worldwide and still pose challenges in response to current treatment strategies. Non-small cell lung cancer (NSCLC) accounts for over 85% of lung cancers diagnosed in the United States and novel therapeutics are needed for the treatment of this disease. First and second generation targeted therapies against specific mutated or rearranged oncogenes in NSCLCs show anti-tumor activity and also increase survival. However, many NSCLC patients eventually develop resistance to these therapies or do not properly respond if they have central nervous system metastases. Thus, this review summarizes recent developments and findings related to the generation of novel targeted therapies recently or currently being developed to tackle hurdles that prior therapies were not able to overcome.

## 1. Introduction

Several signaling pathways and specific oncogenes are deregulated in non-small cell lung cancer (NSCLC) and are known to influence changes linked to tumorigenesis, including apoptosis, proliferation, cell-cycle progression, and gene expression [1]. These key pathways and oncogenes affect the development and progression of NSCLC as well as prognosis and resistance. In fact, the majority of NSCLC patients present advanced stages of the disease and many harbor mutations making them more prone to resistance [2]. Therapies specifically targeting pathways and genes commonly deregulated or mutated in NSCLC are attractive for developing treatments that are optimal in regressing or even curing the disease.

It is crucial to identify novel strategies to combat lung cancers since they account for more cancer-related deaths than breast, prostate, colon, and brain cancers combined, making almost a quarter of all cancer deaths due to lung cancer [3]. More specifically, NSCLC accounts for over 85% of all lung cancer cases in the United States [4]. Most NSCLC patients are not diagnosed until they reached locally advanced or metastatic disease states. In addition, many NSCLC patients experience response failures due to mechanisms of resistance [4].

Strategies focused on inhibiting oncogenic proteins in NSCLC, such as anaplastic lymphoma kinase (ALK), epidermal growth factor receptor (EGFR), c-ros oncogene 1 (ROS1), and v-raf murine sarcoma viral oncogene homolog B1 (BRAF), have been generated and therapies against these proteins have shown promise in combating NSCLC. Despite the development of first generation inhibitors against these oncogenes that showed beneficial responses and effects on survival, these drugs still face challenges related to treating metastasized tumors and avoiding mechanisms of resistance. Thus, new therapies specifically targeting these oncogenes or other components involved in these oncogenic pathways are continually being generated to overcome these hurdles. It is important to mention that along with targeted therapies, immunotherapies, specifically checkpoint inhibitors, have recently showed positive outcomes and strong safety profiles in the treatment of NSCLC [5]. However, in this article, we mainly focus on the progress of targeted therapies for the treatment of NSCLC and the current strategies being undertaken to tackle NSCLC cases with specific mutations or rearrangements since the targeted therapy field has made a substantial progress over recent years. In addition, targeted therapies will most likely be used along with traditional chemotherapeutic strategies and immunotherapies to treat NSCLC in the future [6].

In this review, the rationale behind targeting ALK, EGFR, ROS1, BRAF, and other important targets are summarized based on what has been recently uncovered. In addition, current targeted therapies against these genes/proteins are discussed as well as an overview of how their targeting profiles, ability to overcome resistance and safety have improved or are in the process of being modified for more optimal therapeutic responses.

## 2. ALK Inhibitors

ALK, a component of the insulin receptor tyrosine kinase family, functions in fetal nervous system development but the full extent of its functions are unclear and require further investigation [7,8]. ALK has been shown to combine with fusion partners that lead to its constitutive activation in various cancers, resulting in oncogenic addiction. In NSCLC, ALK fuses with echinoderm microtubule-associated protein-like 4 (EML4) and is also presented in the form of other rearrangements, including fusions with a variety of other genes [9,10]. Altogether, ALK fusions and rearrangements are observed in 2–9% of NSCLC cases and are also associated with specific clinical features, such as a mild to no smoking history [9,10]. Crizotinib, originally developed as a MET inhibitor, is a first generation ALK inhibitor effective in treating NSCLC. It is known to have a response rate of 60% and a median progression-free survival time of 8–10 months [11]. However, despite the successful response rate, many patients relapsed only a few years after starting treatment. In addition, brain metastases in NSCLC patients receiving crizotinib therapy were observed as a consequence of crizotinib’s inability to pass through the blood brain barrier (BBB) [11].

Thus, novel therapies overcoming the issues of resistance as well as potency were needed. Both ceritinib and alectinib are second generation ALK inhibitors that were U.S. Food and Drug Administration (FDA) approved and used to treat NSCLC patients who were previously administered crizotinib [11]. These inhibitors not only proved to be more potent than crizotinib, they also showed to overcome resistance mutations and were effective against brain metastases [11]. For example, ceritinib showed to be effective in both crizotinib-sensitive and crizotinib-resistant ALK-positive NSCLC patients [12]. However, ceritinib showed drawbacks in terms of safety that included thrombotic diseases and respiratory failure [13]. In contrast, alectinib showed a more optimal safety profile than ceritinib. One study investigated *ALK* mutation status before and after treatment with alectinib revealed that the drug remained clinically active against *ALK* rearrangements and mutations as well as against *ALK* variants that could ultimately increase the chances of resistance [14]. In 2018, a third generation ALK inhibitor, lorlatinib, was approved by the FDAthat can pass through the BBB and also exert broad-spectrum potency against resistant mutations that commonly develop during treatment with crizotinib. In addition, lorlatinib is known to have a strong safety profile, with only mild to moderate side effects that are mostly tolerable by patients [15]. A phase 3 CROWN study is currently being performed to analyze whether lorlatinib shows greater efficacy compared to crizotinib as a first-line therapy for the treatment of ALK-positive NSCLCs (Clinicaltrials.gov identifier: NCT03052608). So far, there is data suggesting that the study met its primary endpoint showing that lorlatinib improved progression-free survival when compared to crizotinib (Clinicaltrials.gov identifier: NCT03052608). However, with the risk of acquired mutations promoting resistance, the journey to uncover other novel and effective treatments continues [16].

The second generation ALK inhibitor brigatinib was recently approved by the FDA for the treatment of ALK-positive metastatic NSCLC. A randomized, phase 3 clinical trial ALTA-1L revealed that brigatinib showed significantly longer progression-free survival in NSCLC patients who were not previously treated with an ALK inhibitor compared to patients treated with crizotinib [17]. In addition, increasing the dose of brigatinib shows positive outcomes in patients treated with crizotinib refractory NSCLC [18].

Lastly, a new generation ALK inhibitor known as ensartinib, which is structurally similar to crizotinib, is ten times more potent than crizotinib, shows activity against crizotinib-resistant ALK mutations as well as brain metastasis and is well-tolerated by patients [19]. Patients treated with ensartinib showed double progression-free survival time compared with crizotinib treated patients [20]. In addition, studies have shown that its anti-tumor activity was not influenced by the presence of multidrug resistance transporters [21]. The higher potency, stronger activity overcoming mechanisms of resistance and the tolerability noted in patients makes ensartinib a promising new candidate for optimally treating ALK-positive NSCLCs.

The optimal line of therapy after failure of second-line therapy against ALK positive NSCLCs is currently unknown. In addition to the administration of third generation ALK inhibitors, platinum/pemetrexed-based chemotherapy is also an option. A recent, retrospective study investigated the efficacy of platinum/pemetrexed-based chemotherapy in NSCLC cases refractory to second-generation ALK inhibitors [22]. Findings revealed that platinum/pemetrexed-based therapy showed modest efficacy in ALK-positive NSCLC cases after second-generation therapy failure. It was suggested that platinum/pemetrexed-based therapy should be administered with an ALK inhibitor for a synergistic effect.

There are ongoing clinical trials investigating how a combination of ALK inhibitors and other therapies influence outcomes in treating ALK-positive NSCLC cases. One ongoing trial is studying how alectinib combined with the anti-angiogenic therapy bevacizumab influences both treatment naïve versus previously treated NSCLC patients. This phase III, single arm trial aims to search for safe and effective therapeutic options to treatNSCLC. The study completion date is estimated to be in January 2021 (Clinicaltrials.gov Identifier: NCT03779191).

## 3. EGFR Inhibitors

EGFR is a surface tyrosine kinase belonging to the HER/erbB family of tyrosine kinases that functions in cell proliferation and apoptosis by modulating signaling pathways [23]. Deregulated EGFR results in increased signaling activity that promotes tumor phenotypes, such as invasion, metastasis, angiogenesis, and proliferation [23]. In NSCLC, EGFR is overexpressed in 40–89% of cases and is mutated in 15–20% of patients [23,24,25]. Its high expression and mutation rate in NSCLCs makes EGFR a strong oncogenic target for therapeutic purposes. Another HER/erbB family member, HER2/ERBB2, has also been studied in NSCLC. In addition to actionable ERBB2 amplification (about 2% of NSCLCs), overexpression, and oncogenic mutations (about 3% of NSCLCs), current studies are focusing on ERBB2 phosphorylation, ubiquitination, and internalization rate using novel drugs, such as the antibody–drug conjugate (ADC) ado-trastuzumab emtansine (T-DM1) and trastuzumab deruxtecan (T-DXd) [26].

Both gefitinib and erlotinib are first generation reversible EGFR inhibitors that are standard of care for NSCLC patients harboring *EGFR* mutations [27]. Both inhibitors improve lung and metastatic site lesions in NSCLC patients. Individuals with *EGFR* mutated NSCLC respond well to these inhibitors, which are considered as standard of care for the first year. However, after the first year these patients unfortunately develop resistance that occurs due to the formation of *EGFR* mutations that increase the affinity for ATP binding (T790 mutation) and prevent inhibitor binding [27]. Thus, the development of a second generation of inhibitors was necessary to overcome this challenge.

The goal of second generation EGFR inhibitors, including the irreversible tyrosine kinase inhibitors afatinib and dacomitinib, was to tackle the issue of resistance. However, these inhibitors did not show significant improvements in overall survival at tolerable doses in patients harboring T790 mutations [27]. They did, however, show significant improvements in progression-free survival when compared to standard chemotherapy [28]. These second generation EGFR inhibitors also showed improvements in progression-free survival relative to first generation EGFR inhibitors.

The third generation EGFR inhibitor osimertinib has shown promising results when treating NSCLC patients with T790 mutations that have developed after first-line treatment. It shows superior efficacy treating *EGFR*-mutant NSCLCs when compared with standard, first-line EGFR inhibitors [29]. In addition, it shows a similar safety profile and less adverse events compared to the older generation inhibitors [29] even though it was administered to patients for a longer duration [30]. In general, it exhibits favorable activity in patients with uncommon *EGFR* mutations [31]. It is also important to mention that osimertinib exhibits excellent activity in treatment-naïve advanced NSCLC central nervous system metastases [32]. However, various articles have emphasized that despite its strong therapeutic effects, osimertinib treated patients are still prone to resistance [33,34].

Fortunately, gefitinib has shown to be effective against osimertinib-resistant mutations (Clinicaltrials.gov identifier: NCT03122717). Thus, studies are being performed analyzing the effects of combining osimertinib along with gefitinib in order to combat acquired resistance (Clinicaltrials.gov identifier: NCT03122717). Combining both therapies showed to be tolerable and cleared the *EGFR* mutation from the plasma. The overall survival rate was similar to what was observed in first-line treatments. However, data regarding long-term survival outcomes as well as acquired resistance are currently pending and will shed more light on whether this combination proves to be more beneficial for *EGFR*-mutated NSCLC patients (Clinicaltrials.gov identifier: NCT03122717).

In addition to EGFR inhibitors, combination therapies, including anti-angiogenesis therapies are being considered for the treatment of *EGFR*-mutant NSCLCs. Ramucirumab is a monoclonal immunoglobulin G1 antibody that recognizes the vascular endothelial growth factor (VEGF), a key regulator of tumor angiogenesis that contributes to the development and progression of NSCLC [28]. A randomized, double-blind phase Ib/III study investigating erlotinib combined with ramucirumab for the treatment of previously untreated *EGFR*-mutant metastatic NSCLC was performed. No major toxicities were noted when treating with this combination and encouraging clinical data allow the study to go to phase III. In addition, this study will investigate the efficacy and safety of ramucirumab in combination with gefitinib in previously untreated *EGFR*-mutant NSCLC patients. This study will be completed in 2024 (Clinicaltrials.gov Identifier: NCT02411448). Similar to what was described for ALK inhibitors, several trials have been performed investigating how a combination of bevacizumab with EGFR inhibitors provides clinical benefit to patients with *EGFR*-mutant NSCLCs. Results combining the EGFR inhibitors erlotinib and gefitinib with bevacizumab show promising and beneficial clinical activity [35,36]. NSCLC patients treated with cetuximab, another EGFR inhibitor, exhibited greater overall survival and a greater response in comparison to patients treated with bevacizumab [37]. An ongoing, randomized phase III trial is studying the effects of the chemotherapeutics carboplatin and paclitaxel with or without bevacizumab and/or cetuximab in treating patients diagnosed with stage IV or recurrent NSCLC (Clinicaltrials.gov Identifier: NCT00946712). Lastly, it is important to note that dysregulated redox regulation can also contribute to the pathogenicity of NSCLC by altering EGFR regulation and also contribute to therapy resistance [38]. Doxorubicin, which can generate oxidants, is being tested along with other therapies to treat NSCLC (Clinicaltrials.gov Identifier: NCT03808480).

## 4. ROS1 Inhibitors

ROS1 is a type I integral membrane protein containing tyrosine kinase activity that stimulates signaling pathways involved in cell growth, proliferation, and survival [39]. Rearrangements in *ROS1* promote its fusion with various other genes, leading to constitutive tyrosine kinase activation that in turn triggers the MAPK/ERK, JAK/STAT, and PI3K/AKT/mTOR signaling pathways [39]. It is important to note that rearrangements in *ROS1* are mutually exclusive of other driver mutations known to promote lung oncogenesis [39]. *ROS1* rearrangements account for 1–2% of NSCLC cases and are mostly observed in women, individuals younger than 50 years of age and those who do not have a history of smoking [39].

Since both ROS1 and ALK share a similar homology, crizotinib, a therapy originally generated to target ALK-positive NSCLC was repurposed for treating NSCLCs with *ROS1* rearrangements. A 2014 study published in the New England Journal of Medicine (NEJM) revealed that crizotinib showed significant improvement in antitumor activity in patients diagnosed with advanced *ROS1*-rearranged NSCLC [12]. In an ongoing phase I PROFILE 1001 study, overall survival of ROS1-positive NSCLC patients showed improvements and no adverse side effects were reported [40]. This study is still ongoing with an estimated completion time in 2022 (Clinicaltrials.gov identifier: NCT00585195). Based on the strong efficacy of crizotinib in clinical trials, the FDA approved this drug to be used for the treatment against ROS1-positive NSCLCs. Despite these successful outcomes, resistance still persists as a potential issue for NSCLC patients treated with crizotinib for a prolonged period of time.

Lorlatinib is another tyrosine kinase inhibitor recommended for use after treatment with crizotinib. It is a third generation, potent, and brain penetrant tyrosine kinase inhibitor that shows activity despite the presence of resistance mutations in both *ALK* and *ROS1* [41]. This inhibitor has shown to have strong activity in ROS1-positive NSCLC patients with central nervous system metastases and who were previously treated with crizotinib [41]. This holds a great promise for ROS1-positive NSCLC patients with advanced disease since their therapeutic options are usually very limited.

Similar to the challenges faced when targeting other driver mutations in NSCLC, optimal treatment of ROS1-positive NSCLCs not only requires an inhibitor that can overcome mechanisms of resistance but also one that penetrates through the BBB to treat brain metastases. The ROS1 inhibitor entrectinib was developed to properly penetrate the BBB and remain in the central nervous system as a form of prevention and treatment of metastatic tumors in patients with locally advanced or metastatic ROS1-positive NSCLC [42]. The clinical trial termed ALKA-372-001 revealed that entrectinib effectively controlled disease with a well-tolerated safety profile, suggesting that it can be administered to patients long-term with less adverse side effects [42]. Based on successful clinical trial data, in the summer of 2019, the FDA approved the use of entrectinib for the treatment of metastatic ROS1-positive NSCLC.

With the goal of developing even more potent inhibitors that can cross the BBB and overcome resistance mutations, a number of other drugs have shown activity against ROS1, including brigatinib, cabozantinib, ceritinib, DS-6051b, entrectinib, repotrectinib, and taletrectinib [43]. In fact, both repotrectinib and taletrectinib hold a great deal of promise in being the next generation of ROS1 inhibitors for the treatment of NSCLC. Repotrectinib is a novel next generation tyrosine kinase inhibitor with high potency and selectivity against treatment-naïve and *ROS1* resistance mutant NSCLC [44]. In addition, repotrectinib is able to penetrate the BBB [44]. Altogether, this suggests that it may be used as a first-line treatment or after treatment with other drugs that no longer yield a response [44]. Similarly, taletrectinib was shown to have a high safety profile and exerted activity in patients with crizotinib-refractory ROS1-positive NSCLC [45]. In addition to overcoming the challenges that current ROS1 inhibitors face, studies need to be conducted to determine whether these drugs can be used as a first-line treatment or whether they are better used after treatment with crizotinib. The generation of novel drugs with strong safety profiles and ability to overcome resistance mutations will increase treatment options for ROS1-positive NSCLC patients [43].

## 5. BRAF and MEK Inhibitors

BRAF is a serine/threonine kinase belonging to the RAS/RAF/MEK/ERK pathway and is involved in regulating cell proliferation and growth [46]. Mutations in *BRAF* account for 3.5–5% of NSCLCs and are known to significantly decrease disease-free survival as well as overall survival [46]. Lung cancers with mutant *BRAF* are known to be more aggressive and difficult to treat due to their ability to resist chemotherapy treatment. Half of the mutations found in the *BRAF* gene are the V600E point mutation. Mutations in *BRAF* are more frequent in women and these mutations are commonly found in former or current smokers [46]. Various tyrosine kinase inhibitors have been generated to target this rare but difficult oncogene in NSCLC.

An integrative analysis of *BRAF* nonV600E mutations was performed using genomic profiles of *BRAF*-mutant NSCLCs and a total of 305 unique *BRAF* mutations were identified [47]. The majority of the mutations were missense mutations. Some of these mutations responded to MEK and BRAF inhibitors, whereas others did not show a significant response. This study underlines the importance of recognizing the specific *BRAF* mutations present in NSCLC cases to identify an optimal treatment regimen.

Studies have shown a trend supporting that patients with *BRAF* V600E mutations are less likely to respond to platinum-based chemotherapy treatment compared to NSCLC patients with *BRAF* non-V600E mutations [46,48]. Dabrafenib is an oral selective inhibitor against BRAF. A phase 2, multicenter, nonrandomized, open-label study of untreated and previously treated stage IV, metastatic NSCLC patients harboring *BRAF* V600E mutations were treated with dabrafenib to determine its therapeutic activity [49]. In this trial, patients who were previously administered less therapy better responded to dabrafenib compared with patients who were previously administered multiple lines of therapy [49]. Some serious adverse events were observed using this drug but it showed promise for the treatment of *BRAF* V600E mutant NSCLCs since patients showed a 33% response rate.

Trametinib is a MEK1/MEK2 inhibitor with anticancer activity. A clinical trial was performed combining trametinib with dabrafenib to determine the anticancer effects of simultaneously administering BRAF and MEK inhibitors. A total of 36 out of 57 patients (63%) exhibited a response to the drug combination [50]. This suggested that the combination of trametinib with dabrafenib was a new strategy to achieve high response rates and a manageable safety profile [50]. The activity of the combination appeared to be greater than administration of dabrafenib alone as a monotherapy. Another study was performed to determine how the combination of trametinib and dabrafenib influenced *BRAF* V600E mutant NSCLC patients who were previously untreated [51]. Patients in this clinical trial were given a combination of trametinib and dabrafenib as a first-line treatment. The overall response rate was 64%, where 6% of patients achieved a complete response and 58% achieved a partial response [51]. This trial concluded that the combined therapy showed strong anti-tumor activity and a strong safety profile in *BRAF* V600E mutant NSCLC patients who have not received treatment prior to this study.

Vemurafenib is another BRAF inhibitor thatrecently showed a response rate of 42% in a cohort of NSCLC patients with *BRAF* V600E mutations [52]. Another study was performed to determine whether vemurafenib was effective in both NSCLC patients with *BRAF* V600E mutations and NSCLC patients with *BRAF* nonV600E mutations. Conclusions from this study revealed that vemurafenib was only effective in treating NSCLC patients with *BRAF* V600E mutations and was not influential in treating NSCLC patients with *BRAF* nonV600E mutations. This study suggested that this drug could only be used in cases where BRAF showed the V600E point mutation and that routine biomarker screener should contain an analysis for BRAF mutations [53].

Despite the promising results supporting these BRAF inhibitors in the fight against NSCLC, the challenge of resistance still persists. One recent study investigated resistance mutations forming as a result of prolonged treatment with trametinib, dabrafenib, and vemurafenib [54]. MEK, KRAS, NRAS, and PTEN mutations were identified as molecular hits in patients treated with BRAF inhibitors and were uncovered as potentially new molecular events promoting resistance. These data supported that the MAPK pathway is reoccurring in *BRAF* mutant treated NSCLCs and these findings should guide the development of novel strategies around tumor resistance. Another study focused on how the development of epithelial-mesenchymal transition (EMT) results in a fluid landscape that may explain rapid disease progression and the lack of response to treatment [55]. While new studies continue to uncover the mechanisms behind resistance, new investigational drugs need to be uncovered that can be used in combination with current treatments or as second or third-line strategies

Lifirafenib is an investigational, reversible BRAF V600E inhibitor as well as an inhibitor of A-RAF, B-RAF, C-RAF, and EGFR that was studied in a phase I, dose-escalation trial investigating the safety and efficacy of the drug in *BRAF* mutant as well as *RAS* mutant sold tumor patients [56]. Responses were observed in most patients tested and future studies may be performed identifying the effectiveness of lifirafenib compared with first generation BRAF therapies and how this drug works in combination with other BRAF or RAS inhibitors.

Additional, ongoing studies are being performed to investigate how some of these BRAF inhibitors work in combination with other therapies to promote a synergistic, anti-tumor effect in NSCLC patients. One study currently being performed by MD Anderson Cancer Center includes a combination of trametinib and pembrolizumab, an immunotherapy, to identify the response in NSCLC patients with recurrent, locally advanced or metastatic disease. This study is estimated to be completed Fall of 2020 (Clinicaltrials.gov identifier: NCT03225664). Other inhibitors targeting the RAS/RAF/MEK/ERK axis are also being investigated as potential therapies to treat NSCLCs with *BRAF* mutations. A phase I study of the RAF inhibitor LXH254 is currently being performed in patients with advanced solid tumors that have alterations in the MAPK pathway (Clinicaltrial.gov Identifier: NCT02607813). This study is aimed to be completed in Spring of 2021.

Since BRAF is a component of the MAPK pathway, it is important to note that there are other therapeutics being generated to target other aspects of this pathway, such as inhibitors against the proto-oncogene MET that encodes for the hepatocyte growth factor receptor and plays a major role in embryogenesis [57]. Various drugs are being tested for clinical activity against MET-positive NSCLCs since it is overexpressed in approximately 20% of NSCLCs and correlates with prognosis. For one, crizotinib has shown strong activity in 30–40% of MET amplified or mutated NSCLC cases [58]. Some studies show a strong response to crizotinib while others also show strong survival benefit [59]. In addition, crizotinib appears to be effective in the treatment of *EGFR*-mutated NSCLCs consisting of MET amplifications, which contribute acquired resistance in about 10% of *EGFR*-mutated NSCLC cases [60,61,62].

The MET inhibitor capmatinib was shown to be highly selective and potent [63]. In addition to showing an increased survival benefit, it also exerts activity on the central nervous system and has been shown to be a promising therapeutic choice after the failure of crizotinib [64]. Capmatinib has also shown to be effective in the treatment of NSCLC patients harboring MET Exon 14 skipping mutations that occur in 4% of all NSCLC cases, as well as patients containing MET amplifications [63]. Another MET inhibitor known as tepotinib has been approved by the FDA for advanced NSCLCs containing MET mutations. This inhibitor is highly potent, selective and exerts central nervous system-penetrant activity [65]. It also showed a partial response in half of advanced NSCLC patients containing *MET* Exon 14 skipping mutations [65]. Tepotinib along with the EGFR inhibitor osimertinib are be tested in combination to determine whether these two drugs have synergistic anti-tumor activity on MET-positive NSCLCs and the study is estimated to be completed in 2023 (Clinicaltrials.gov identifier: NCT03940703). Other promising inhibitors against MET-positive NSCLCs also being investigated in clinical trials include the experimental small molecular MET inhibitor savolitinib and the small molecular MET inhibitor cabozantinib. In a clinical trial expected to be completed next year, savolitinib is being administered to locally advanced or metastatic NSCLC patients with *MET* Exon 14 mutations (Clinicaltrials.gov identifier: NCT02897479). A multicenter, single arm, phase II study planned to be completed Fall of 2020 is evaluating the efficacy of cabozantinib treatment in a NSCLC cohort harboring MET amplifications or mutations (Clinicaltrials.gov identifier: NCT03911193).

## 6. Additional Oncogenic Targets

In recent years, new oncogenic drivers of NSCLC that can be targeted for therapeutic purposes have been uncovered. Some of these novel drivers include neurotrophic receptor tyrosine kinases (NRTKs), neuregulin-1 (*NRG1*) gene fusions and rearranged during transfection (*RET*) gene fusions that are commonly observed in NSCLC cases. First, the functions of these genes will be briefly described. Then, a quick overview of some promising therapies targeting these drivers in NSCLC will be summarized.

NRTKs are typically involved in neural development and are found to be deregulated in various solid tumors, including NSCLCs. The frequency of NRTK fusions in NSCLCs is relatively low (less than 0.5%) [57]. When NRTKs fuse with other genes, they become constitutively active and stimulate various signaling cascades, including the MAPK and PI3K pathways. In 2018 and 2019, both larotrectinib and entrectinib received approval for the treatment of NRTK-fusion positive cancers. Larotrectinib exerted marked and durable anti-tumor activity in most TRK-positive cancers despite differences in age or tumor type [66]. Similarly, entrectinib was found to have a durable and clinically meaningful response in TRK-positive solid tumors as well as a nicely tolerated safety profile [67].

NRG1 is a ligand member of the EGFR family that has been shown to be genetically rearranged in about 0.5% of NSCLCs [57]. When rearranged and fused to specific fusion partner genes, the HER2/HER3 signaling cascades and PI3K/AKT/mTOR as well as RAS/MAPK signaling pathways are stimulated [57]. Inhibitors against HER2/HER3 are currently being tested to tackle NRG1-positive NSCLCs. The irreversible pan-HER inhibitor afatinib has shown to have clinical activity in NSCLC patients with NRG1-positive tumors [68]. Clinical trials are currently being performed to continue investigating the potential of afatinib in tackling NRG1-positive NSCLCs (Clinicaltrials.gov identifier: NCT02925234). Additionally, the HER3 inhibitor GSK2849330 has also shown a durable response and greater activity when compared with afatinib in patients with NRG1-positive NSCLC cancer [57,69].

RET is a transmembrane receptor tyrosine kinase with oncogenic characteristics and the ability to signal the PI3K/AKT, JNK, and RAS/MAPK pathways [57]. If genetic rearrangements occur in *RET*, these pathways are continually activated and lead to tumorigenesis. Genetic rearrangements and translocations in *RET* have been identified in about 1% of all lung cancers [70]. Interestingly, the incidence of *RET* rearrangements is higher in never smokers, women and those with poorly differentiated tumors [57].

Some studies are investigating how the use of drugs originally generated for other tyrosine kinase inhibitors can be repurposed to tackle RET-positive NSCLCs. In one study, patients with RET-positive NSCLC were recruited from 29 different centers around the world and they received one or more different tyrosine kinase therapies [71]. This retrospective study found that already available tyrosine kinase inhibitors only showed limited effectiveness in RET-rearranged NSCLC patients. Thus, novel therapies are needed to tackle this subpopulation of NSCLC cases.

Fortunately, the selective RET inhibitors selpercatinib and pralsetinib have demonstrated more substantial anti-tumor activity in NSCLC patients harboring *RET* rearrangements [57]. Selpercatinib showed durable efficacy, intracranial activity, and less toxic effects in RET-fusion positive NSCLC patients [72]. Similarly, pralsetinib demonstrated durable, potent, and broad anti-tumor activity in NSCLC patients with advanced RET-fusions (Clinicaltrials.gov identifier: NCT03037385). Future studies and clinical trials will shed light onto what drug is most optimal for the treatment of *RET*-rearranged NSCLC cases and what combinations of other tyrosine kinases or chemotherapies should be used to ensure that a patient achieves an optimal response.

## 7. Conclusions and Future Directions

When specific oncogenes are mutated or rearranged, they can contribute to the formation or progression of NSCLC. Over the last decade, much progress has been made in generating novel therapeutics to target some of these oncogenes. This review provides a summary of targeted therapies that were generated, tested, and are either still currently used for the treatment of NSCLC or set aside to make room for better drugs with greater potency, safety, and activity against both the primary and metastasized tumors. Some of these drugs have been recently in clinical trials (Table 1) and are being tested as sole or combination therapies for the treatment of NSCLC. As briefly mentioned, it is important to emphasize that immunotherapies (including checkpoint inhibitors and vaccines) as well as angiogenesis therapies (VEGF therapies) are also at the forefront of NSCLC treatment. There are currently two immunotherapies used to treat NSCLC that target the cytotoxic T-lymphocyte antigen-4 protein (CTLA-4), including ipilimumab and tremelimumab. In addition, the checkpoint PD-1 inhibitors nivolumab and pembrolizumab as well as the PD-L1 inhibitor atezolizumab are used as second-line treatments for NSCLC [73]. In addition, there are a variety of studies interrogating the beneficial effects of combining immunotherapies with other inhibitors, such as gefitinib [74] and cyclo-oxygenase inhibitors [75]. However, here we focus on targeted therapies since the road for these therapies has been long and much improvement has been made in their development over the years. It will be interesting to observe how systemic therapy continues to alter based on the identification and understanding of specific biomarkers over the next few years [76].

Even though several candidate oncogenes targeted to treat NSCLC have been uncovered and extensively studied, we are still in search of others that can generate stronger therapeutic responses. Similar to a dart board, we have been throwing darts to tackle targets already uncovered by developing newer and better therapies against those specific target genes. However, identifying additional targets, perhaps unknown oncogenes that, if targeted, will provide a strong therapeutic response, will bring us many steps closer to treating this disease. Many of the treatment strategies discussed in this article are very promising, indicating that we are on the right path to unlocking the best therapies that can be used in the future.

## Figures and Tables

**Table 1:** Summary of selected targeted therapies that have been in clinical trials recently for the treatment of NSCLC.

Trial Identifier	Target(s)	Trial Title
NCT03052608	ALK, ROS1	A Study Of Lorlatinib Versus Crizotinib In First Line Treatment Of Patients With ALK-Positive NSCLC
NCT03779191	ALK	Alectinib in Combination With Bevacizumab in ALK Positive NSCLC
NCT03122717	EGFR	Osimertinib and Gefitinib in EGFR Inhibitor naïve Advanced EGFR Mutant Lung Cancer
NCT02411448	VEGFR2, EGFR	A Study of Ramucirumab (LY3009806) in Combination With Erlotinib in Previously Untreated Participants With EGFR Mutation-Positive Metastatic NSCLC (RELAY)
NCT00585195	MET, ALK, ROS1	A Study Of Oral PF-02341066, A C-Met/Hepatocyte Growth Factor Tyrosine Kinase Inhibitor, In Patients With Advanced Cancer (PROFILE 1001)
NCT03225664	MEK	Trametinib and Pembrolizumab in Treating Patients With Recurrent Non-small Cell Lung Cancer That Is Metastatic, Unresectable, or Locally Advanced
NCT02607813	RAF	Phase I Study of LXH254 in Patients With Advanced Solid Tumors Harboring MAPK Pathway Alterations
NCT03940703	MET	A Study of Tepotinib Plus Osimertinib in Osimertinib Relapsed Mesenchymal-epithelial Transition Factor (MET) Amplified Non-small Cell Lung Cancer (NSCLC) (INSIGHT 2)
NCT02897479	MET	A Phase II Study of HMPL-504 in Lung Sarcomatoid Carcinoma and Other Non-small Cell Lung Cancer
NCT03911193	MET	CABozantinib in Non-Small Cell Lung Cancer (NSCLC) Patients With MET Deregulation (CABinMET)
NCT02925234	multiple, NRG1	The Drug Rediscovery Protocol (DRUP Trial) (DRUP)
NCT03037385	RET	Phase 1/2 Study of the Highly-selective RET Inhibitor, Pralsetinib (BLU-667), in Patients With Thyroid Cancer, Non-Small Cell Lung Cancer, and Other Advanced Solid Tumors (ARROW)
